# Dual-Mode, Orientation-Adaptive Broadband Rotational Energy Harvester for Diverse Noise and Vibration Environments †

**DOI:** 10.3390/mi17070775

**Published:** 2026-06-26

**Authors:** Md Shamim Ahmed, Xianghong Ma, Yu Jia

**Affiliations:** College of Engineering and Physical Sciences, Aston University, Birmingham B4 7ET, UK; x.ma@aston.ac.uk (X.M.); y.jia1@aston.ac.uk (Y.J.)

**Keywords:** rotational energy harvesting, dual-mode operation, nonlinear bi-stability, orientation adaptation, broadband vibration

## Abstract

Rotational energy harvesters are often constrained by narrow operating bandwidths and sensitivity to specific rotational regimes, limiting their effectiveness under variable-speed conditions. This work presents an orientation-adaptive dual-mode piezoelectric rotational energy harvester capable of broadband energy extraction across diverse rotational and vibration environments. The proposed design combines gravity-induced magnetic excitation at low rotational speeds with centripetal-force-induced nonlinear dynamics at higher rotational speeds, enabling passive transition between operating modes without active tuning. A coupled nonlinear electromechanical model is developed to investigate the interactions among gravitational forcing, magnetic coupling, centripetal loading and piezoelectric transduction. Numerical simulations reveal the transition from gravity-dominated mono-stable behaviour to broadband nonlinear operation as rotational speed increases. Experimental validation is conducted using representative vibration profiles from aerospace, automotive, civil infrastructure and industrial environments. The results demonstrate clear orientation-dependent performance, with the downward cantilever configuration achieving a maximum average power output of 57.8 μW under aerospace elevation excitation, whilst the upward configuration exhibits improved robustness under broadband random vibrations. The proposed orientation-adaptive framework provides a compact, stator-independent solution for broadband rotational energy harvesting under realistic operating conditions.

## 1. Introduction

Rotational energy harvesting has emerged as a compelling strategy for enabling self-powered sensing and monitoring in systems where continuous or intermittent rotational motion is inherently available, including aerospace platforms, automotive wheels, wind and turbine machinery, and industrial rotating equipment [1]. Compared to translational vibration energy harvesting, rotational harvesting offers access to large and persistent kinetic energy; however, effective conversion of this energy into usable electrical power remains challenging due to strong dependence on angular velocity and excitation characteristics. A preliminary version of this work was presented in [2]. The present article substantially extends that conference paper through additional theoretical modelling, expanded numerical and experimental investigations, revised discussions, and enhanced analysis.

A fundamental limitation of most reported rotational energy harvesters is their narrow operational angular velocity range. In real environments, rotational speed is rarely constant and often varies significantly due to changes in operating conditions, load variations, start–stop cycles, and external disturbances. Harvesters optimised for a single rotational regime therefore exhibit degraded performance or complete loss of functionality outside their design range, severely limiting their applicability for long-term autonomous operation.

Low-speed rotational energy harvesters typically rely on gravity-induced excitation, compliant oscillators, pendulum-like mechanisms, or weak inertial perturbations to activate the transduction element [3,4,5]. These approaches are effective when centripetal acceleration is negligible; however, as angular velocity increases, the rapidly growing centripetal force introduces strong static bias and axial loading. This effect suppresses transverse motion, shifts equilibrium positions, and often forces the oscillator into a locked or quasi-static state, dramatically reducing energy conversion efficiency [6]. As a result, many gravity- or inertia-driven designs exhibit a sharp performance drop once rotational speed exceeds a modest threshold.

Conversely, rotational energy harvesters designed for high angular velocities—including rotor–stator electromagnetic generators and gyroscopic systems—are capable of efficient energy extraction under rapid rotation [7,8]. Nevertheless, these devices generally require rigid axial coupling, precise alignment, and the presence of a fixed stator reference. Such requirements impose significant constraints on integration and render these systems unsuitable for parasitic energy scavenging, where structural modification is undesirable or where no stationary reference frame is available. Moreover, generator-type systems often suffer from increased mechanical complexity, wear, and maintenance demands, further limiting their deployment in distributed sensing applications.

This dichotomy gives rise to a persistent design gap in rotational energy harvesting: most existing devices perform well either at low or at high angular velocities but lack the adaptability required for broadband operation across widely varying rotational regimes. In many practical applications—such as vehicle wheels, wind turbine blades, or rotating machinery—rotational speed may transition from slow roll to rapid spin within a single duty cycle [1,9]. Under such conditions, single-mode harvesters are unable to provide sustained and reliable power output, motivating the development of adaptive or multi-regime harvesting strategies.

Recent studies have demonstrated that nonlinear dynamics, particularly bi-stability and multi-stability, can significantly enhance the bandwidth and robustness of vibration energy harvesters by enabling amplitude-dependent inter-well oscillations [4,6]. Magnetically induced bi-stability has been shown to be especially effective due to its tunability, contactless nature, and strong nonlinearity. However, the majority of nonlinear rotational harvesters reported in the literature remain limited to a single operational regime and do not explicitly address the transition between gravity-dominated low-speed dynamics and centripetal-force-dominated high-speed dynamics.

Recent advances in piezoelectric energy harvesting have further demonstrated the effectiveness of magnetically coupled transduction mechanisms for self-powered monitoring applications. For example, Clementi et al. reported a hybrid energy harvesting approach that combines multiple energy sources to improve the reliability and sustainability of autonomous sensing systems, highlighting the growing importance of advanced energy harvesting strategies in practical engineering applications [10]. These developments further support the use of nonlinear and magnetically assisted mechanisms for improving energy extraction under variable operating conditions.

To overcome these limitations, this work proposes a dual-mode rotational energy harvester based on a piezoelectric cantilever with a magnetic tip mass and a freely orbiting permanent magnet. The system is deliberately engineered to exploit different physical mechanisms across distinct rotational regimes. At low angular velocities, gravity-assisted excitation of the free magnet produces rotation-synchronous magnetic forcing of the cantilever, enabling effective energy harvesting without reliance on resonance. As angular velocity increases, centripetal acceleration introduces axial loading that destabilises the upright cantilever configuration, activating a magnetically induced nonlinear bi-stable regime. In this high-speed mode, amplitude-dependent inter-well oscillations enable broadband response and enhanced energy conversion under ambient vibrational disturbances.

Furthermore, the harvester incorporates orientation-adaptive piezoelectric cantilever configurations, allowing passive exploitation of gravitational bias and inertial effects under diverse noise environments. This orientation dependence enables improved robustness against non-stationary and broadband excitations commonly encountered in aerospace, automotive, and civil infrastructure applications. By allowing a single compact structure to transition autonomously between mono-stable and bi-stable dynamic regimes, the proposed design overcomes the conventional trade-off between low- and high-speed performance, providing a stator-independent and broadband solution for rotational energy harvesting under realistic operating conditions.

Despite significant progress in rotational and nonlinear vibration energy harvesting, most existing systems are designed to operate efficiently within a limited rotational regime or under specific excitation conditions. Gravity-assisted rotational harvesters are generally effective at low rotational speeds, whereas nonlinear bi-stable harvesters are primarily developed for broadband vibration environments. Furthermore, the influence of cantilever orientation under realistic rotational and noise-driven operating conditions remains insufficiently explored. Consequently, a gap exists for a unified energy harvesting framework capable of adapting to both low- and high-speed rotational regimes whilst maintaining robust performance under diverse broadband excitations. To address this challenge, this work proposes an orientation-adaptive dual-mode rotational energy harvester that combines gravity-induced excitation, nonlinear magnetic coupling, and centripetal-force-induced dynamics within a single compact architecture.

## 2. Unified Harvester Architecture and Operating Principles

**Figure 1:**
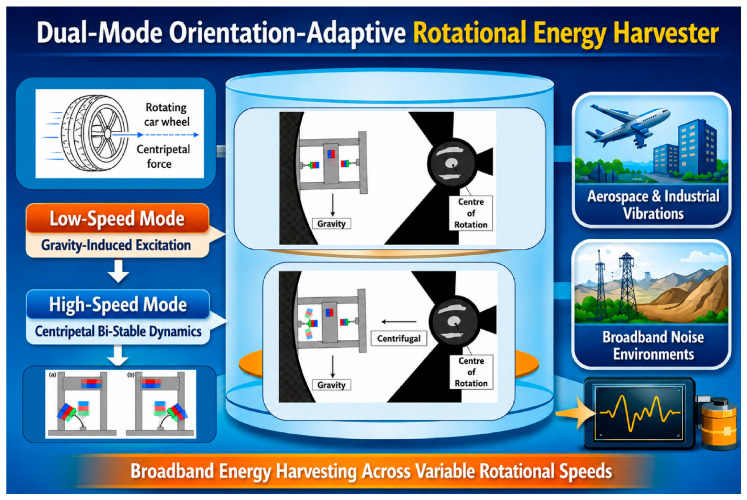
Graphical representation of proposed dual-mode orientation-adaptive rotational energy harvester.

The rotational energy harvester investigated in this work is developed as a unified electromechanical platform that integrates multiple excitation mechanisms within a single compact structure. Building upon the progressive development reported in earlier studies [11,12], the architecture is intentionally designed to exploit gravity-induced excitation, magnetic coupling, centripetal-force-driven stiffness modulation, and orientation-dependent dynamics, enabling broadband energy harvesting across wide angular velocity ranges and diverse vibration environments (Figure 1). Rather than treating low-speed and high-speed rotational harvesting as separate design problems, the present system allows a single physical structure to transition passively between distinct dynamic operating regimes as rotational speed and excitation conditions vary. This unified approach eliminates the need for active tuning, mechanical reconfiguration, or a fixed stator reference, which are common limitations in conventional rotational energy harvesters.

### 2.1. System Architecture

The core architecture consists of one or more piezoelectric cantilever beams equipped with permanent magnetic tip masses, magnetically coupled to a freely moving permanent magnet constrained within a guiding channel. The cantilever beams are mounted in an upright orientation with respect to the centre of rotation, such that their longitudinal axes are aligned radially. This configuration is deliberately selected to suppress static transverse locking at high rotational speeds while enabling axial inertial loading effects under centripetal acceleration [13,14]. Magnetic coupling between the cantilever tip masses and the free magnet is configured to be repulsive, introducing a strongly nonlinear, distance-dependent interaction force. This non-contact coupling replaces mechanical plucking or impact-based excitation mechanisms, thereby eliminating wear and enabling tunable nonlinearity through magnet strength, spacing, and geometry. The free magnet is permitted to translate under the combined influence of magnetic forces, gravity, and inertial effects arising from rotation [2,15].

**Figure 2:**
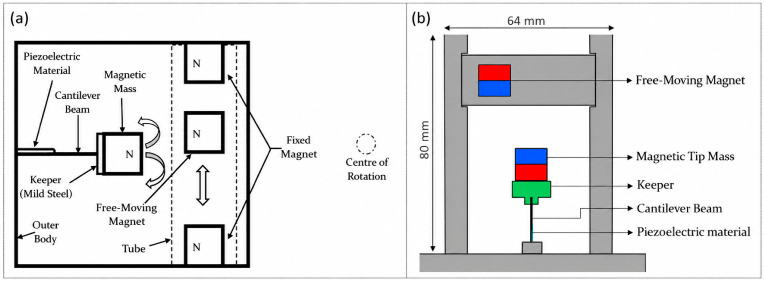
(**a**) Schematic representation of the magnetically coupled cantilever–magnet system operating in a rotating frame [2] and (**b**) the structural configuration of the compact piezoelectric rotational energy harvester with an upright cantilever and free-moving magnet [15].

Across the different design iterations reported in [1,2,3,4,5], this architecture is extended to include upward- and downward-orientated cantilever configurations, enabling orientation-adaptive behaviour under realistic vibration environments. The overall system is compact and lightweight, minimising added inertia and imbalance when mounted on rotating components such as wheels, blades, or shafts, and enabling parasitic energy scavenging without structural modification. A conceptual overview of the unified harvester architecture and magnetic coupling mechanism is provided in Figure 2.

The free-moving magnet was constrained to move along the vertical axis of the guide tube under the combined influence of gravity, magnetic interaction, and rotationally induced inertial forces. The magnetic separation distance was selected to provide strong nonlinear coupling whilst avoiding magnetic locking between the free-moving magnet and the magnetic tip mass. This configuration enabled both gravity-assisted excitation at low rotational speeds and nonlinear dynamic behaviour under increased rotational loading. The geometric and physical parameters used in the proposed harvester are summarised in Table 1. 

#### 2.1.1. Advantages of Rotational Configuration

Conventional cantilever energy harvesters typically rely on translational base excitation and operate efficiently only within a limited frequency range around resonance. In contrast, the proposed rotational architecture introduces two additional physical mechanisms: gravity-induced excitation and rotational-speed-dependent centripetal loading. At low rotational speeds, gravitational modulation of the free-moving magnet generates periodic magnetic forcing of the cantilever, enabling energy harvesting without resonance. As rotational speed increases, centripetal loading modifies the effective stiffness of the cantilever and, in conjunction with magnetic repulsion, alters the potential energy landscape of the system. This process enables a passive transition from mono-stable to bi-stable dynamics, thereby broadening the operational bandwidth. Consequently, the rotational geometry provides capabilities that are not readily achievable using a conventional fixed cantilever configuration.

#### 2.1.2. Governing Equations and Dynamic Model

The dynamic behaviour of the proposed rotational energy harvester is governed by the coupled interaction between the free-moving magnet, the piezoelectric cantilever, gravitational excitation, magnetic forces, and rotationally induced inertial effects. The equations of motion presented below are adapted from our previously validated nonlinear dynamic model and are included here to clarify the physical mechanisms responsible for passive mode switching and broadband energy harvesting.

The equation for the free-moving magnet is given below:

(1) $$m_{m}\ddot{y}\;+\;c\dot{y_{m}}\;+a{\left|y\left(t\right)\right|}_{-y}^{+y}=\;N\left(t\right)+mg\sin\left(\omega t+\eta_{0}\right)$$

where y is the displacement of the free-moving magnet, $m_{m}$ is its mass, c is its damping coefficient, a is the magnetic-force-generated linear coefficient, N(t) is the external force applied to the system due to road roughness noise or other sources and $mg\sin\left(\omega t+\eta_{0}\right)$ is the rotation of the entire system.

The dynamic behaviour of nonlinear vibration systems is strongly influenced by the interaction between system parameters, excitation characteristics, and phase-dependent response mechanisms. Recent studies have demonstrated the importance of accurately characterising dynamic response deviations and nonlinear interactions when analysing complex vibration systems under varying operating conditions [16]. Motivated by these considerations, the governing equations of the proposed rotational energy harvester are presented below.

Equation (2), an approximate representation of the dipole interaction between two magnets, may be used to express the magnetic spring force.

Here,

(2) $$F_{mag}(y,t)=-\frac{\pi {µ}_{0}M^{2}r^{4}}{4}\left[\frac{1}{{y\left(t\right)}^{2}}+\frac{1}{{\left(y\left(t\right)+2h\right)}^{2}}+\frac{2}{{\left(y\left(t\right)+h\right)}^{2}}\right]$$

where $F_{mag}$ is the magnetic force, ${µ}_{0}$ is the permeability of free space (1.26 × 10^−6^ H/m), *M* is the magnetisation field (*M* = *B*/*µ*_0_), *B* stands for the Tesla flux density, *r* stands for the magnet’s radius, *h* for its thickness, *y* for its displacement, *d* for its separation from the other magnets, and *t* for the time domain.

Furthermore, the stiffness affects the oscillator’s critical damping (*c_c_*). Critical damping, however, behaves like stiffness, which is a displacement-dependent phenomenon. The constant stiffness *k*, natural frequency $\omega_{n}(y,t)$, critical damping $c_{c}$ and damping constant c can be expanded as shown in Equations (3) and (6), respectively,

(3) $$k=\frac{F_{mag}(y,t)}{y(t)}$$

(4) $$\omega_{n}(y,t)=\sqrt{\frac{F_{mag}(y,t)}{my(t)}}$$

(5) $$c_{c}(y,t)=2m\sqrt{\frac{F_{mag}(y,t)}{my(t)}}$$

(6) $$c=2\zeta m\sqrt{\frac{F_{mag}(y,t)}{my(t)}}$$

Also, the linear coefficient produced from the magnetic force is defined as

(7) $$a=\mu_{0}v^{2}\frac{9M_{cx}M_{fx}-12M_{cy}M_{fy}}{4\pi d^{7}}$$

Here, a is the magnetic-force-generated linear coefficient, v is the volume of the magnet, d is the distance between two magnets, and $M_{cx},\;M_{fx},\;M_{cy}\;and\;M_{fy}$ are the magnetisation amplitudes.

(8) $$N\left(t\right)=\sqrt{2D}\zeta (t)$$

In addition, $N\left(t\right)$ is the noise, D is the intensity of noise and ζ(t) is the Gaussian white noise with time domain. And $mgsin\left(\omega t+\eta_{0}\right)$ is the rotation where g is the gravitational acceleration, the rotational frequency is ω, and the initial phase angle is $\eta_{0}$.

The equation for the motion of the upper left cantilever beam:

(9) $$m\ddot{x}\;+\;c\dot{x_{m}}\;+\;\left(k-\frac{3m\omega^{2}H}{2L}-a(y,t)\right)x-{bx}^{3}=\;N\left(t\right)+mg\sin\left(\omega t+\eta_{0}\right)$$

where the displacement of the cantilever beam is x, its mass is m, the damping coefficient is c, the spring constant is k, a is the coefficient of the linear term of the magnetic bar’s restoring force, the coefficient of the cubic is b in terms of the magnetic bar’s restoring force, H is the distance between the centre of rotation and a cantilever beam, L is the length of the cantilever beam, N(t) is the external force applied to the system due to road roughness noise or other sources, and $mg\sin\left(\omega t+\eta_{0}\right)$ is the rotation of the entire system.

Also, the magnetic force produces a nonlinear coefficient defined as

(10) $$b=\mu_{0}v^{2}\frac{75M_{cx}M_{fx}-90M_{cy}M_{fy}}{8\pi d^{7}}$$

Here, b is the nonlinear produced by the magnetic-force coefficient, v is the volume of the magnet, d is the distance between two magnets, and $M_{cx},\;M_{fx},\;M_{cy}\;and\;M_{fy}$ are the magnetisation amplitudes.

The coefficients (a) and (b) govern the effective linear and nonlinear restoring forces of the system. As the rotational speed increases, the centripetal-force-induced stiffness modification interacts with the nonlinear magnetic restoring force, causing the potential energy landscape to evolve from a single-well (mono-stable) configuration to a double-well (bi-stable) configuration. This transition forms the basis of the passive mode-switching mechanism observed in the proposed harvester.

### 2.2. Low-Speed Gravity-Dominated Operating Regime

At low angular velocities, centripetal acceleration remains small relative to gravitational acceleration, and system dynamics are dominated by gravity-induced effects. In this regime, the freely moving magnet behaves as a gravity-biased inertial mass whose equilibrium position varies continuously with the rotational angle of the device [17]. As rotation progresses, the direction of gravitational force relative to the rotating frame reverses every half rotation, producing periodic modulation of the free magnet position [18]. This gravitational modulation generates a time-varying magnetic force acting on the cantilever tip mass, effectively exciting the cantilever through non-contact magnetic plucking. The excitation is synchronised with rotational motion rather than with the cantilever’s natural frequency, enabling energy harvesting under slow, quasi-static, or irregular rotation. In this operating regime, the cantilever dynamics are predominantly mono-stable, with oscillation amplitude governed by the balance between gravitational forcing, magnetic coupling strength, elastic restoring force, and structural damping. This gravity-dominated mode forms the primary energy harvesting mechanism during slow rotational motion, startup conditions, or intermittently rotating systems. The resulting dynamic response characteristics and associated energy conversion performance are analysed quantitatively through numerical simulations and experimental measurements in later sections.

### 2.3. High-Speed Centripetal-Induced Bi-Stable Operating Regime

**Figure 3:**
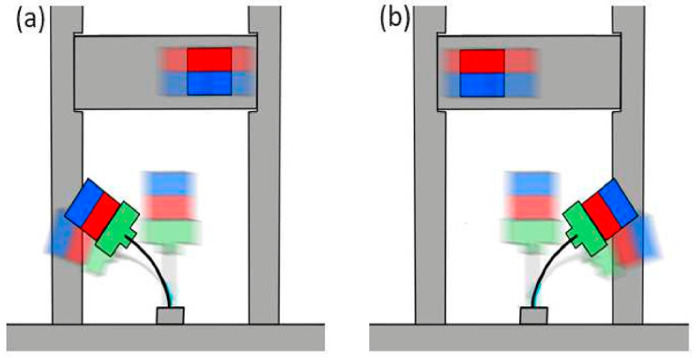
Schematic illustration of REH operating (**a**) intra-well positive and (**b**) intra-well negative and achieving a bi-stable system [15].

As angular velocity increases, centripetal acceleration scales quadratically with rotational speed and becomes the dominant inertial force acting on the system. In the upright cantilever configuration, this acceleration introduces an effective axial compressive load along the beam, reducing its effective linear stiffness and altering the system’s potential energy landscape [19]. When combined with the repulsive magnetic interaction between the cantilever tip mass and the free magnet, this axial stiffness reduction gives rise to a nonlinear bi-stable potential characterised by two stable equilibrium positions separated by an unstable equilibrium [20]. Unlike mechanically buckled or geometrically complex bi-stable structures, this bi-stability emerges naturally from the interaction between centripetal-force-induced stiffness modulation and magnetic nonlinearity (Figure 3). In this high-speed regime, ambient vibrations, rotational disturbances, or broadband noise can provide sufficient energy to overcome the potential barrier, triggering snap-through motion between the two stable states. These inter-well oscillations are inherently amplitude-dependent and enable broadband frequency response, allowing effective energy harvesting across a wide range of excitation frequencies. Once snap-through motion is initiated, centripetal acceleration further amplifies the dynamic response by accelerating the cantilever deeper into each potential well, increasing strain energy in the piezoelectric layer and enhancing electrical output. This centripetal-induced bi-stable regime underpins the harvester’s ability to operate effectively at high rotational speeds, where gravity-driven mechanisms become ineffective. The nonlinear dynamics and broadband harvesting characteristics associated with this mode are demonstrated through numerical and experimental results presented in subsequent sections.

### 2.4. Orientation-Adaptive Behaviour Under Diverse Noise Environments

**Figure 4:**
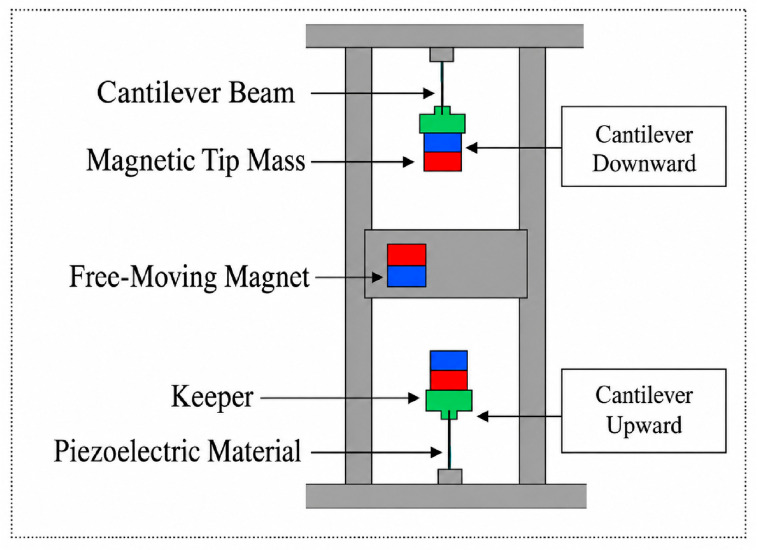
Graphical representation of proposed REH.

Extending beyond purely rotational considerations, the unified architecture incorporates upward and downward cantilever orientations (Figure 4) to exploit gravitational bias and inertial asymmetry under realistic vibration environments [21,22]. The orientation of the cantilever relative to gravity and excitation direction significantly influences the probability of inter-well transitions, effective bandwidth, and harvested power under non-stationary and broadband noise. This orientation-adaptive behaviour allows the same harvester architecture to respond differently to aerospace, automotive, civil infrastructure, and industrial vibration environments, providing an additional passive mechanism for performance optimisation without active control. The influence of cantilever orientation on dynamic response and energy harvesting performance is systematically investigated in the experimental sections of this paper.

Polyvinylidene fluoride (PVDF), a lightweight and flexible piezoelectric polymer, has been widely applied for vibration-based energy harvesting owing to its durability and ease of integration with mechanical structures. Previous research investigated composite-based nonlinear vibration energy harvesters and highlighted their superior broadband response. A research paper [23] further explored miniature rotational harvesters, demonstrating the role of cantilever orientation in power output. However, experimental studies explicitly comparing cantilever-upward and downward REHs under diverse environmental noise conditions remain limited. This gap motivates the present work, which provides systematic testing under aerospace in-flight noise, aerospace elevation noise, bridge vibrations, road-car engine vibrations, and compressor-induced noise. These environments represent practical operational contexts, thus ensuring this study’s relevance to real-world applications.

Energy harvesting from vibration in aerospace platforms presents an attractive solution for powering distributed sensor nodes in locations where battery replacement is impractical. Rotational energy harvesters (REHs) that use piezoelectric materials offer a compact and passive approach. However, one challenge is capturing energy across a broad frequency spectrum, especially under variable aerospace operating conditions. This study evaluates a broadband REH design that employs both upward and downward cantilever configurations to extract energy from rotation-induced vibrations in aerospace environments [21].

The increasing electrification of modern aircraft has intensified the demand for reliable and lightweight onboard power solutions. Current battery technologies, such as Li-Ion and Ni-Cd, present several drawbacks: they add mass, require frequent reconditioning or recharging, and create environmental concerns due to hazardous disposal processes [24]. Harvesting vibration energy provides a more sustainable solution, as it converts mechanical vibrations into usable electrical energy. Piezoelectric harvesters are a promising option for vibration-based energy harvesting: previous aerospace uses have explored piezoelectric patches mounted on the fuselage, wing roots, and leading slats, but these locations often show limited vibration amplitudes, which reduces their energy harvesting potential. The landing gear represents an underexplored site for energy harvesting. During taxiing, take-off, and landing, the landing gear is subject to significant vibrational amplitudes. Harnessing this energy could reduce reliance on batteries for powering onboard sensors, such as accelerometers, strain gauges, and temperature monitors, which are essential for predictive maintenance and health monitoring [25].

**Figure 5:**
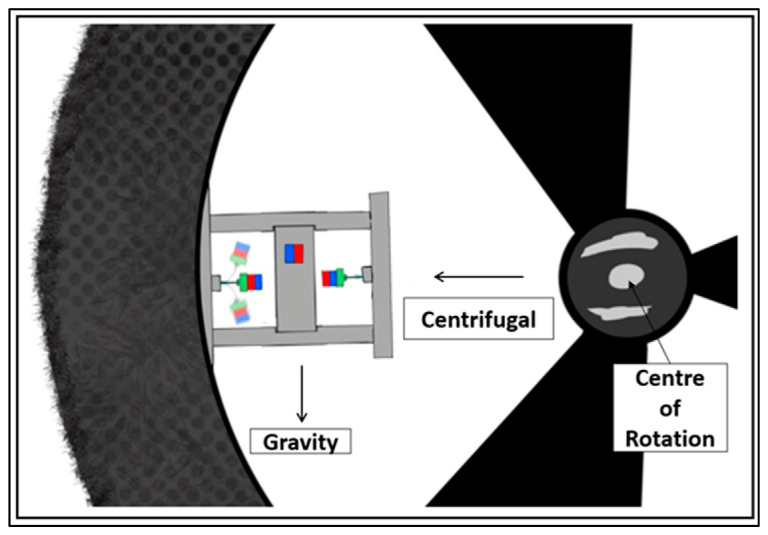
Cantilever dynamic response under centrifugal loading.

**Figure 6:**
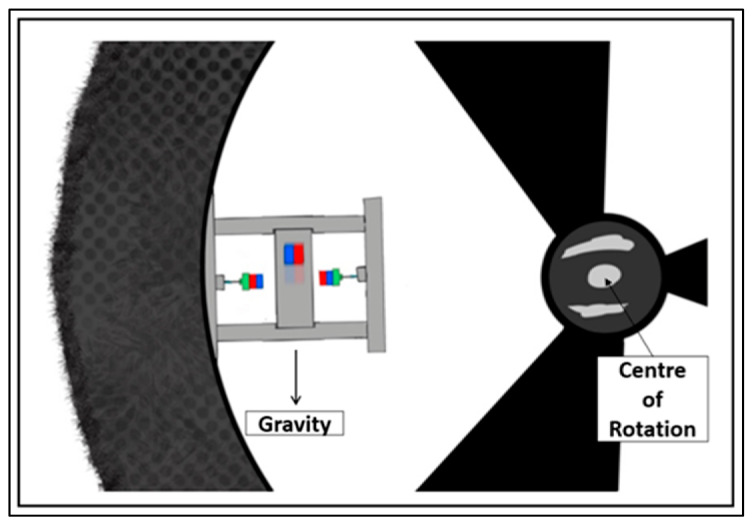
Gravitational regime showing mono-stable upward and downward cantilever behaviour.

Figure 5 and Figure 6 illustrate the operational behaviour of the proposed dual-mode piezoelectric harvester under both rotational and low-speed gravitational environments. In the rotational case (Figure 4), the centrifugal field increases radially outward from the centre of rotation and introduces a significant downward inertial load on the structure. This force biases the system toward a cantilever-downward mono-stable configuration, as the centrifugal stiffening effect suppresses upward bending motion and effectively constrains the beam to a single stable equilibrium in the downward direction. Although the cantilever upward direction is inherently capable of exhibiting bi-stable behaviour due to the nonlinear magnetic interaction between the tip magnet and the free-moving magnet, this bi-stability is prevented at high rotational speeds because the free magnet becomes forced to one extreme side of its enclosure. As a result, the system behaves as mono-stable in both the upward and downward directions under sufficiently high angular velocity, with the upward potential well effectively collapsed by centrifugal confinement.

In contrast, the low-speed gravitational operating regime shown in Figure 5 reveals a different dynamic response. When the rotational speed is small and centrifugal forces become negligible, both the cantilever-upward and cantilever-downward directions behave as mono-stable configurations determined primarily by gravity and ambient vibration. In this case, the free-moving magnet is no longer locked to one side and instead oscillates vertically in response to gravity. This vertical motion produces a time-varying magnetic-force gradient, which in turn modulates the effective stiffness experienced by the cantilevers in both directions. Thus, even though the beams remain mono-stable at low speed, their displacement is significantly influenced by magneto-mechanical coupling.

Unlike conventional linear energy harvesters that rely on resonance within a narrow frequency range, the proposed harvester exploits nonlinear bi-stable dynamics to interact effectively with broadband excitation. Under random vibration environments containing multiple frequency components, the system can undergo both intra-well oscillations and inter-well snap-through transitions. These nonlinear transitions redistribute energy across a wider frequency range and generate higher-order dynamic responses, enabling effective energy harvesting from broadband noise environments. Consequently, the harvester does not depend on a single resonant frequency and is capable of extracting energy from diverse vibration spectra commonly encountered in aerospace, automotive, civil infrastructure, and industrial applications.

## 3. Results and Discussion

The numerical simulations were performed using the nonlinear dynamic model presented in Section 2.1.2. The free-moving magnet was constrained to translate only along the guide channel under the combined influence of gravity, magnetic interaction, rotational loading, and external excitation. The cantilever was modelled as a lumped single-degree-of-freedom system, whilst the magnetic interaction was represented using the dipole-based approximation described in Equation (2). Material properties and damping characteristics were assumed to remain constant throughout the simulations, and the external excitation was represented by Gaussian white noise with a prescribed intensity. Air resistance, thermal effects, and material ageing were neglected to focus on the dominant electromechanical behaviour of the harvester. Gravitational acceleration was assumed constant throughout the rotational cycle.

The principal parameters governing the system response include the magnetic coupling strength, cantilever stiffness, damping ratio, rotational speed, and the separation distance between interacting magnets. These parameters influence the effective potential energy landscape of the system and determine the transition between mono-stable and bi-stable operating regimes. The optimisation strategy focused on achieving stable broadband response, avoiding magnetic locking, and promoting inter-well oscillations under broadband excitation. Particular attention was given to maximising the probability of snap-through motion while maintaining stable operation and avoiding excessive cantilever deformation. These considerations formed the basis for the numerical investigations presented in the following sections.

### 3.1. Numerical Demonstration of Dual-Mode Operation

Numerical simulations are performed to establish the fundamental feasibility of the proposed dual-mode rotational energy harvester and to elucidate the nonlinear dynamic mechanisms governing its operation across different angular velocity regimes. The numerical model is derived from the coupled nonlinear equations of motion previously developed in [2,15], incorporating gravitational excitation, magnetic dipole interaction, structural elasticity, viscous damping, and centripetal-force-induced axial loading. This modelling framework enables systematic investigation of the influence of rotational speed on system stability and dynamic response prior to experimental validation.

**Figure 7:**
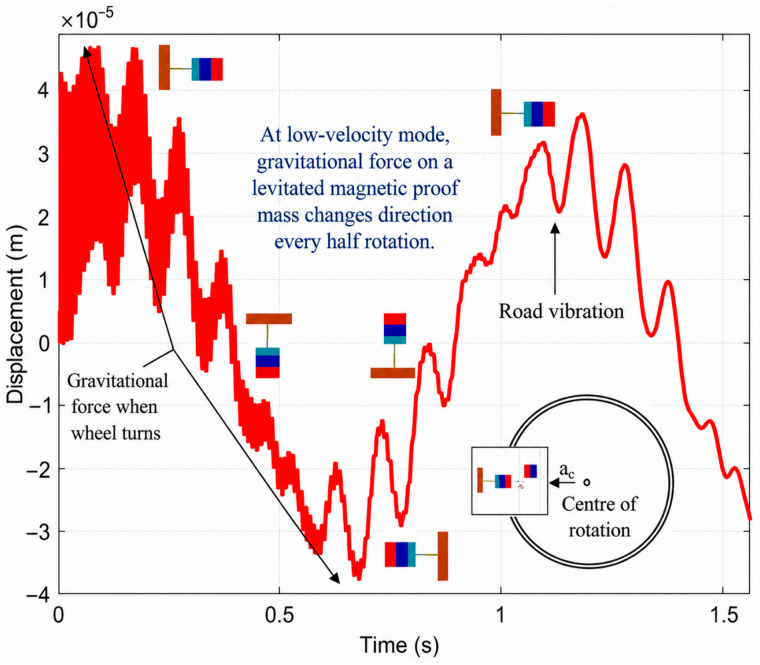
Gravity-assisted mono-stable cantilever response at low rotational speed.

At low angular velocities, the simulated time-domain response of the piezoelectric cantilever exhibits bounded oscillations about a single stable equilibrium position, indicating mono-stable behaviour. In this regime, centripetal acceleration is small compared with gravitational acceleration, and the system dynamics are dominated by gravity-induced motion of the freely moving magnet. As the device rotates, the gravitational force acting on the free magnet reverses direction every half rotation, producing a periodic modulation of the magnetic interaction force applied to the cantilever tip mass. This rotation-synchronous magnetic excitation drives cantilever oscillations without reliance on resonance, while the restoring force remains effectively linear. A representative numerical time-history illustrating gravity-dominated mono-stable dynamics is shown in Figure 7. These results confirm that, at low rotational speeds, the harvester operates in a gravity-assisted mono-stable mode consistent with the operating principles proposed in [2].

**Figure 8:**
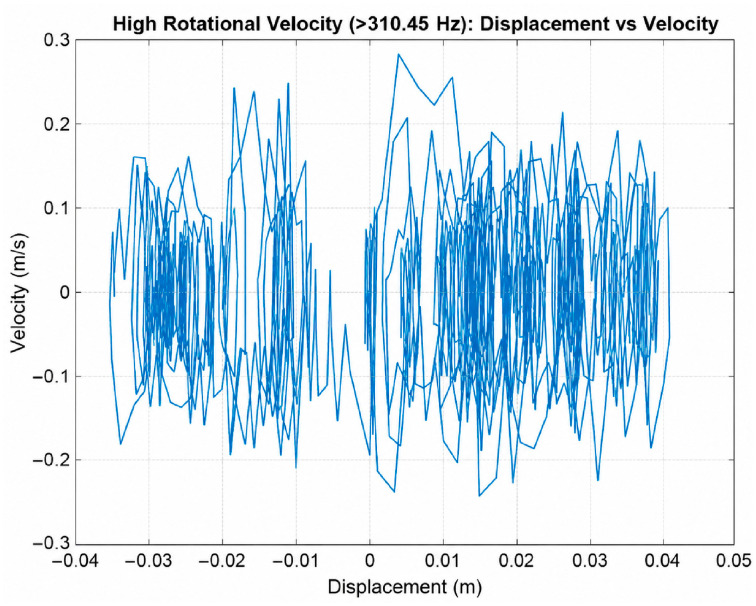
Displacement–velocity phase portrait of the cantilever response at elevated rotational velocity, illustrating nonlinear oscillatory behaviour under centripetal-force-dominated conditions.

As angular velocity increases, a pronounced qualitative change in system behaviour is observed. The centripetal acceleration, which scales quadratically with rotational speed, introduces a significant axial compressive load along the upright cantilever. This axial loading reduces the effective linear stiffness of the beam and, when combined with the repulsive magnetic coupling between the cantilever tip mass and the free magnet, leads to a deformation of the system’s potential energy landscape. Beyond a critical rotational speed, the single equilibrium configuration bifurcates into two stable equilibrium positions separated by an unstable equilibrium, giving rise to a nonlinear bi-stable system. Numerical simulations in this regime reveal large-amplitude oscillations accompanied by intermittent snap-through motion between the two potential wells, as illustrated in Figure 8. This behaviour is indicative of centripetal-force-induced bi-stability and is absent at low rotational speeds [2,15,26].

It should be noted that Figure 8 represents a phase-space trajectory rather than a single-valued displacement function. In phase space, the velocity corresponding to a particular displacement depends on both the direction of motion and the dynamic state of the system. Consequently, the same displacement position may be associated with different velocity values during different stages of the oscillation cycle. In the proposed harvester, this behaviour is further influenced by magnetic nonlinearity and rotational effects, which modify the energy exchange process and produce complex phase-space trajectories. The observed multi-valued displacement–velocity relationship is therefore characteristic of nonlinear oscillatory systems and provides evidence of the broadband dynamic behaviour exploited for energy harvesting.

Importantly, the numerical results demonstrate that the transition from mono-stable to bi-stable dynamics occurs passively as a function of angular velocity, without any change in system geometry or configuration. Magnetic coupling plays a critical role in enabling stable oscillatory behaviour across both regimes: at low speeds it facilitates gravity-driven excitation through non-contact plucking, while at high speeds it contributes to the formation of a well-defined bi-stable potential that prevents static locking under strong centripetal loading. These numerical results demonstrate that the proposed harvester exhibits distinct dynamic regimes as a function of angular velocity, with gravity-dominated mono-stable behaviour at low speeds and centripetal-force-induced bi-stability at elevated speeds.

### 3.2. Nonlinear Bi-Stable Dynamics and Broadband Frequency Characteristics

At elevated angular velocities, the dynamic response of the proposed rotational energy harvester is governed by nonlinear effects arising from the combined influence of centripetal-force-induced axial loading and magnetically mediated restoring forces. Numerical analysis is employed to characterise the resulting bi-stable dynamics, with emphasis on phase-space structure, intra- and inter-well motion, and the associated broadband frequency characteristics.

As demonstrated in Section 3.1, increasing angular velocity introduces a substantial axial compressive load along the upright cantilever due to centripetal acceleration. This axial load reduces the effective linear stiffness of the beam and, when combined with repulsive magnetic coupling between the cantilever tip mass and the freely moving magnet, leads to a deformation of the system’s potential energy landscape. Beyond a critical rotational speed, the single-well potential bifurcates into a double-well configuration containing two stable equilibrium positions separated by an unstable equilibrium. This nonlinear potential structure forms the basis of the observed bi-stable dynamics and is consistent with the magnet-induced bi-stability mechanisms reported in earlier studies.

**Figure 9:**
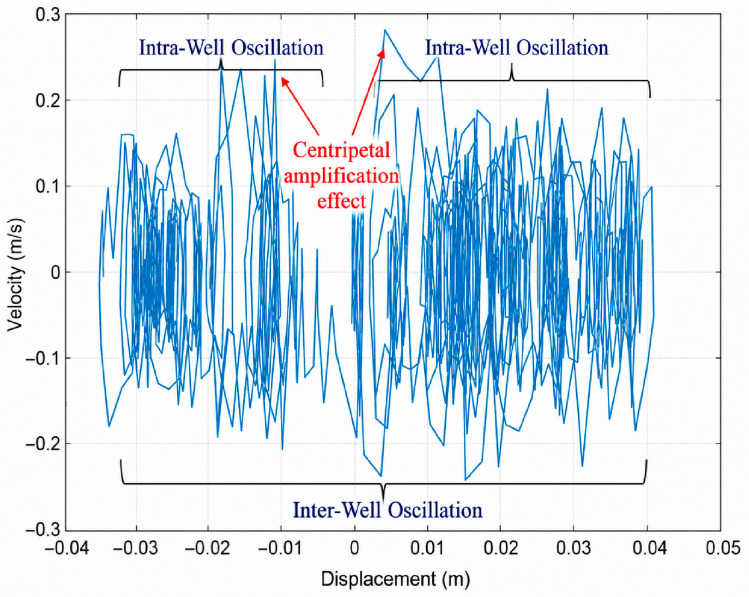
Interpreted phase-space representation highlighting intra-well and inter-well oscillations and the centripetal amplification effect in the bi-stable operating regime.

The nonlinear dynamic response in the bi-stable regime is illustrated through the displacement–velocity phase portrait shown in Figure 9. The response exhibits both intra-well oscillations, characterised by closed phase-space trajectories localised around individual equilibrium positions, and inter-well snap-through motion, manifested as trajectories traversing between the two potential wells. The coexistence of these responses reflects the strongly nonlinear nature of the system and highlights the sensitivity of the dynamic state to excitation amplitude and initial conditions.

**Figure 10:**
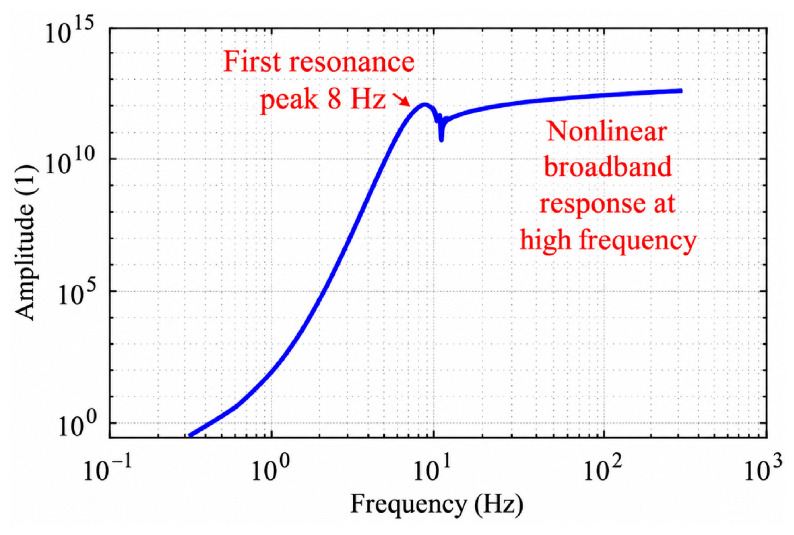
Broadband frequency characteristics of the cantilever response under bi-stable operation.

The frequency characteristics associated with bi-stable operation differ fundamentally from those observed in the gravity-dominated mono-stable regime. Frequency-domain analysis of the cantilever response reveals that intra-well oscillations are associated with relatively narrow spectral content centred around an effective natural frequency modified by axial loading. In contrast, once inter-well snap-through motion is activated, the response spectrum broadens significantly, with energy distributed across a wide range of frequencies and higher-order harmonics, as shown in Figure 10. This broadband frequency response arises from the non-periodic, amplitude-dependent nature of snap-through motion and is a defining feature of bi-stable energy harvesters [2].

As shown in Figure 10, the first resonance peak occurs at approximately 8 Hz. Beyond this region, the response remains elevated across a broad frequency range, indicating the influence of nonlinear dynamics and demonstrating the broadband characteristics of the proposed harvester.

Magnetic coupling plays a central role in shaping the nonlinear response and ensuring stable oscillatory behaviour in the bi-stable regime. The repulsive magnetic interaction not only contributes to the formation of the double-well potential but also prevents static collapse of the cantilever under strong centripetal loading. Moreover, asymmetry introduced by magnetic interaction and gravitational bias can distort the potential wells, influencing the likelihood and directionality of inter-well transitions. This asymmetry is reflected in the phase-space trajectories and contributes to the robustness of broadband response under varying excitation conditions [27].

The numerical results presented in this section demonstrate that centripetal-force-induced bi-stability fundamentally alters the system dynamics, enabling broadband frequency characteristics that are unattainable in conventional linear or purely gravity-driven rotational energy harvesters. These nonlinear dynamic features provide the physical basis for enhanced energy harvesting performance at high rotational speeds and underpin the experimental investigations of bi-stable behaviour and broadband response presented in the subsequent sections.

### 3.3. Experimental Studies Under Controlled Laboratory Excitation

Experimental investigations were conducted to validate the dual-mode operating behaviour predicted by the numerical analysis and to demonstrate the practical feasibility of the proposed rotational energy harvester under controlled laboratory excitation. The experimental study builds on the prototype design and testing methodology reported in [15], with particular emphasis on verifying gravity-dominated operation at low effective angular velocities and the emergence of nonlinear bi-stable dynamics under increased excitation intensity. The experiments were designed to isolate the dynamic response of the harvester and to assess its ability to transition between operating regimes without mechanical reconfiguration or active control.

**Figure 11:**
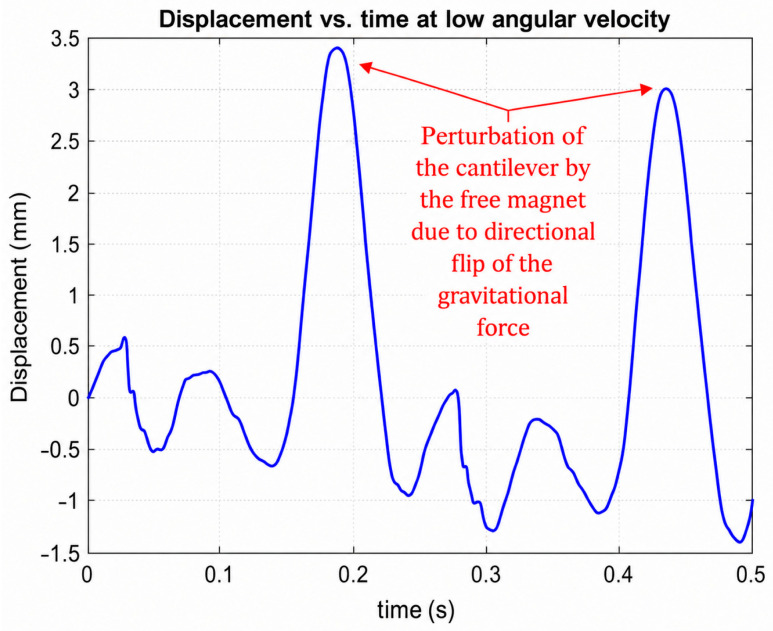
Displacement vs. time indicating gravitational mode at low angular velocities.

Figure 11 presents the measured displacement–time response of the cantilever under low angular velocity excitation, corresponding to the gravity-dominated operating regime. In this condition, the system exhibits stable and repeatable periodic oscillations, indicating mono-stable behaviour. The observed response arises from the gravitational force acting on the freely moving magnet, which reverses direction during each half cycle of rotation. This periodic change in gravitational loading produces a time-varying magnetic interaction force on the cantilever tip mass, effectively plucking the cantilever through non-contact magnetic coupling. As a result, the cantilever vibrates about a single equilibrium position with bounded amplitude, confirming the intended gravitational operating mechanism and demonstrating effective energy conversion under low-speed rotational conditions.

**Figure 12:**
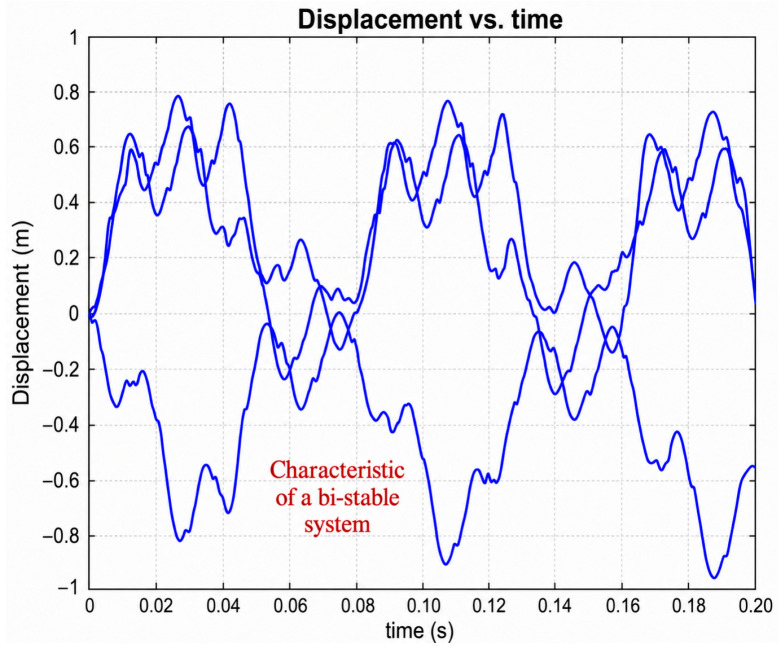
Displacement vs. time indicating bi-stability at high angular velocities.

**Figure 13:**
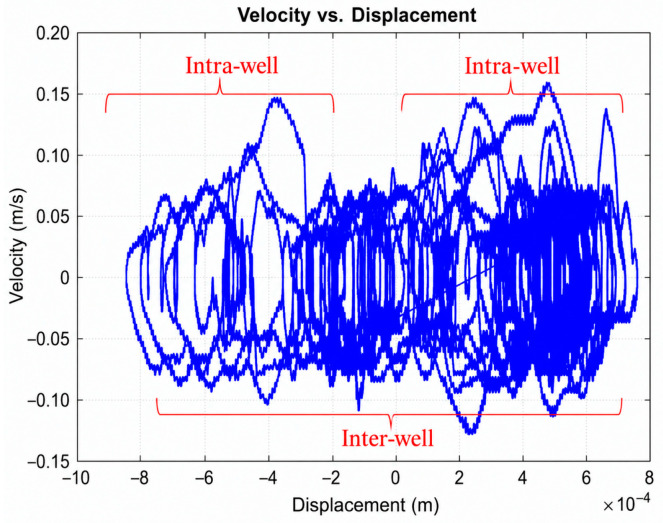
Velocity vs. Displacement confirming two stable equilibrium points.

As the excitation intensity is increased to emulate higher rotational speeds, a clear qualitative change in the system response is observed. The displacement–time response shown in Figure 12 exhibits large-amplitude oscillations with irregular temporal characteristics, indicating the onset of nonlinear dynamics. In contrast to the low-speed case, the response no longer remains confined to a single equilibrium position. Instead, abrupt transitions between two preferred deflected states are observed, suggesting the activation of bi-stable behaviour driven by increased inertial loading and magnetic nonlinearity. These large-amplitude oscillations are accompanied by a significant increase in mechanical strain, which directly enhances the electromechanical energy conversion process. Further evidence of bi-stable dynamics is provided by the velocity–displacement phase portrait shown in Figure 13. The presence of distinct closed loops in the phase space indicates the existence of two stable equilibrium points, while trajectories traversing between these loops correspond to inter-well snap-through motion. The coexistence of intra-well oscillations and intermittent inter-well transitions reflects the strongly nonlinear nature of the system and confirms the combined influence of centripetal effects and repulsive magnetic interaction in shaping the system’s potential energy landscape. These experimentally observed phase-space characteristics are consistent with the bi-stable dynamics predicted numerically and provide direct experimental validation of gravity- and magnet-induced bi-stability.

**Figure 14:**
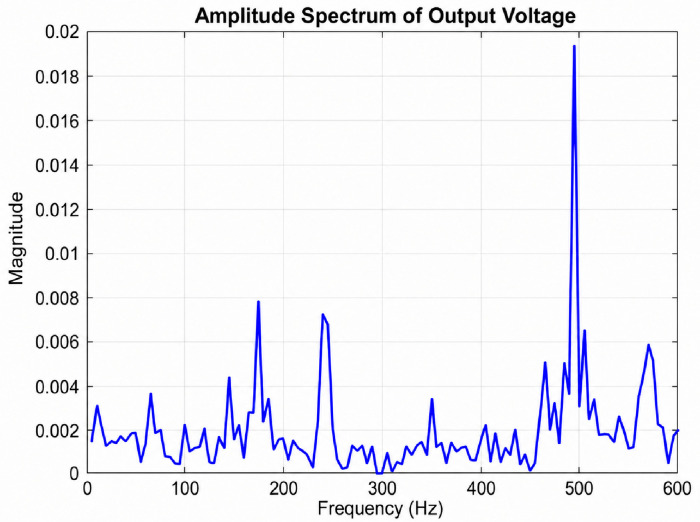
Measured output voltage amplitude spectrum of the proposed rotational energy harvester.

Figure 14 presents the measured output voltage amplitude spectrum of the proposed rotational energy harvester. A dominant frequency component is observed near 500 Hz, together with several lower-amplitude spectral components distributed across the measured frequency range. The voltage signals were acquired directly using a PicoScope 5000 Series oscilloscope without digital filtering. The figure is intended to provide a qualitative representation of the measured electrical response under the investigated operating conditions.

Overall, the experimental results demonstrate good qualitative agreement with numerical predictions, confirming the existence of distinct gravity-dominated and bi-stable operating regimes within a single compact structure. Minor discrepancies between numerical and experimental responses, such as variations in oscillation amplitude and transition thresholds, can be attributed to structural damping, material losses in the PVDF layer, magnetic asymmetry, and alignment tolerances inherent in the experimental setup. Despite these practical non-idealities, the experiments provide strong experimental evidence supporting the proposed gravity- and magnet-induced bi-stability concept and confirm the capability of the harvester to operate effectively across a wide range of angular velocities, addressing a key limitation of existing rotational energy harvesting technologies [15].

### 3.4. Orientation-Adaptive Performance Under Diverse Noise Environments

**Figure 15:**
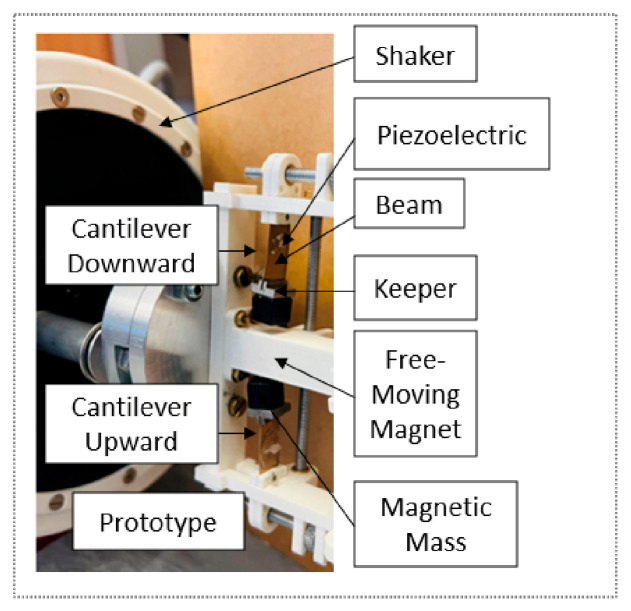
Experimental setup of proposed REH.

To evaluate the robustness and practical applicability of the proposed rotational energy harvester under realistic operating conditions, experimental investigations are extended to a range of non-stationary vibration environments representative of real-world applications. Building on the controlled laboratory validation presented in Section 3.3, this study examines the influence of cantilever orientation on energy harvesting performance under diverse noise profiles, including aerospace in-flight vibration, aerospace elevation manoeuvres, civil infrastructure vibration, automotive engine excitation, and industrial machinery noise [21,28,29]. Two cantilever configurations are considered: an upward-orientated cantilever and a downward-orientated cantilever, enabling assessment of orientation-dependent dynamic behaviour arising from gravitational bias and inertial asymmetry (Figure 15).

**Figure 16:**
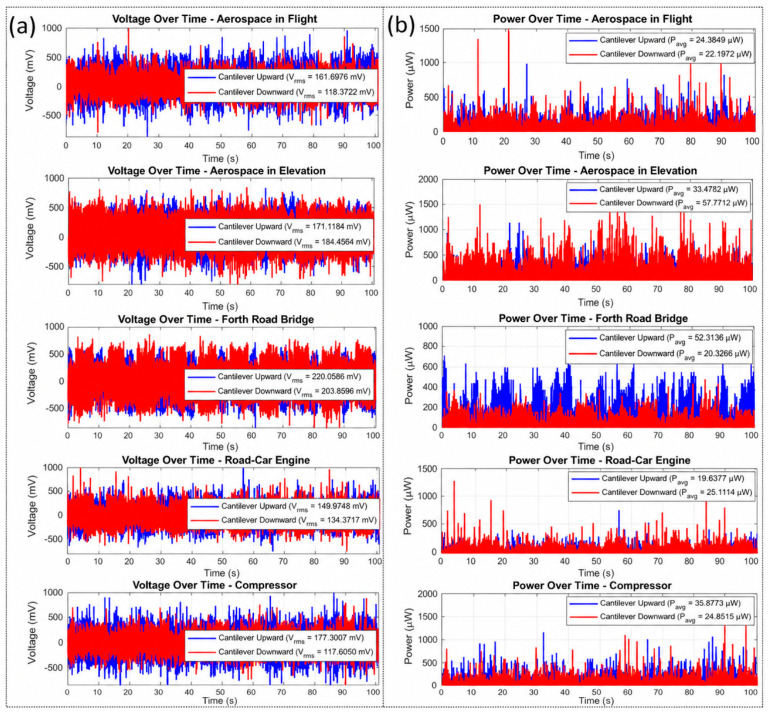
(**a**) Measured output voltage responses of the upward and downward cantilever configurations under five representative vibration environments: aerospace in flight, aerospace in elevation, Forth Road Bridge, road-car engine, and compressor. (**b**) Corresponding output power responses of the upward and downward cantilever configurations under the same vibration environments.

Figure 16 presents the time-domain responses of the upward and downward cantilever configurations under representative aerospace vibration profiles. Distinct differences in response amplitude and temporal characteristics are observed between the two orientations. Under relatively stable and moderate-amplitude excitation, such as aerospace in-flight vibration, the upward-orientated cantilever exhibits more consistent voltage output, indicating improved alignment between the excitation spectrum and the effective dynamic response of the cantilever. In contrast, under more energetic and broadband excitation associated with aerospace elevation manoeuvres, the downward-orientated cantilever produces significantly higher voltage amplitudes, suggesting enhanced activation of nonlinear dynamics due to favourable gravitational bias.

**Figure 17:**
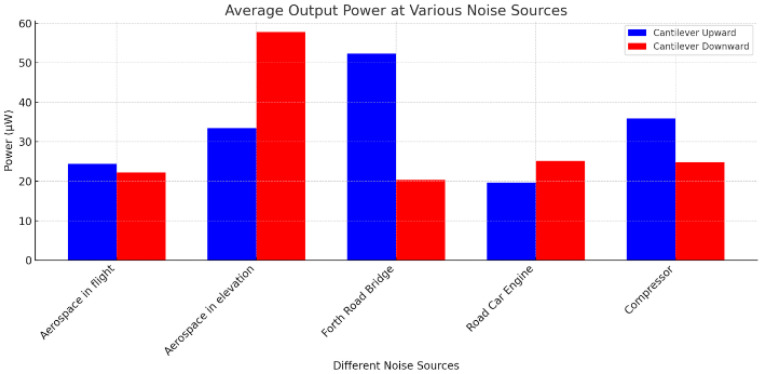
Average output power at various noise sources.

In Figure 17, the results reveal a clear orientation-dependent performance trend. While the upward cantilever demonstrates superior performance under lower-amplitude and more stationary excitation conditions, the downward cantilever consistently outperforms under high-energy and broadband vibration environments, achieving peak average power output of up to 57.8 µW under elevation-type excitation [28]. This complementary behaviour indicates that cantilever orientation plays a critical role in governing the probability of snap-through events and the effective utilisation of available vibrational energy.

**Figure 18:**
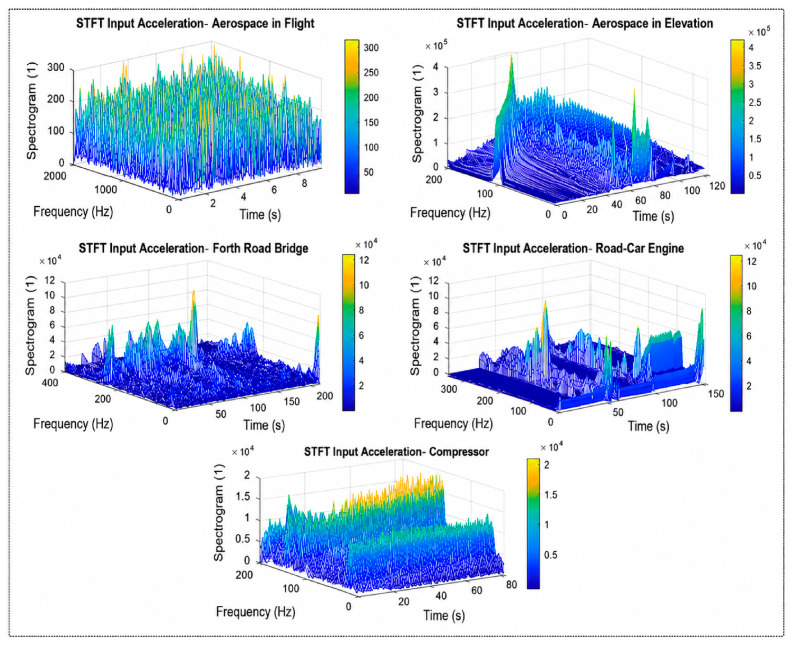
STFT input acceleration of different noise environments.

To further elucidate the underlying mechanisms driving orientation-adaptive behaviour, time–frequency analysis based on short-time Fourier transform (STFT) is employed. Figure 18 shows the STFT representations of the input acceleration signals and the corresponding voltage responses for selected excitation cases. The analysis reveals a strong correlation between broadband frequency content in the excitation and enhanced energy harvesting performance, particularly for the downward-orientated cantilever. In this configuration, gravitational bias lowers the effective energy barrier for inter-well transitions, increasing the likelihood of snap-through motion under transient high-energy components of the excitation. Conversely, the upward-orientated cantilever exhibits reduced sensitivity to such transient components but provides a more stable response under narrower-band excitation.

The orientation-dependent performance observed in these experiments highlights the importance of passive adaptability in rotational energy harvesting systems operating under non-stationary and unpredictable excitation. Rather than relying on active tuning or complex control strategies, the proposed architecture exploits gravitational and inertial asymmetry to achieve environment-dependent optimisation of energy harvesting performance. This orientation-adaptive capability enables the harvester to respond differently to distinct vibration spectra, enhancing robustness across a wide range of applications.

**Figure 19:**
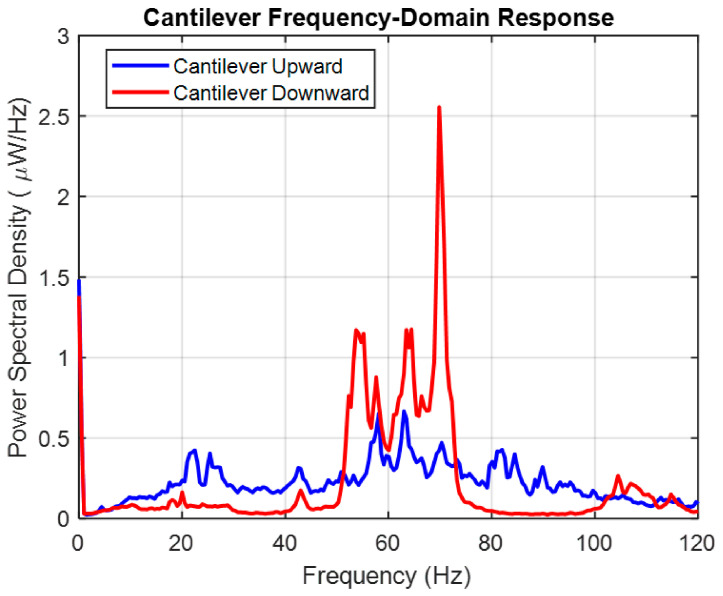
Frequency-domain response indicates broadband.

The frequency domain shows the broadband rotational energy harvester (Figure 19). Both orientations are effective but environment-specific: the downward configuration excels in elevation-type vibration dominated by vertical loading, whereas the upward design is broadly effective across flight, civil infrastructure, automotive, and industrial environments. RMS voltage and average power measurements, supported by STFT evidence, confirm the robustness of the nonlinear magnet–cantilever interaction and establish the proposed design for vibration-powered applications in aerospace, automotive, and structural health monitoring contexts.

Although the proposed harvester demonstrated effective energy harvesting performance under the investigated vibration environments, several practical considerations should be noted. The present prototype employs PVDF as the piezoelectric transduction material due to its flexibility, low density, and ease of integration. However, prolonged exposure to elevated temperatures may influence its piezoelectric properties and mechanical stiffness. The experimental investigations reported in this study were conducted under laboratory conditions and did not include dedicated thermal ageing or high-temperature testing. Consequently, the reported results are representative of moderate-temperature operating environments. For deployment in high-temperature automotive or industrial applications, thermal protection measures or alternative piezoelectric materials with higher thermal stability may be required. Evaluation of long-term thermal reliability will be considered in future work.

The vibration profiles employed in this study were used as representative broadband excitation environments to compare the relative performance of the proposed harvester under different operating conditions. The focus of this investigation was on the comparative energy harvesting behaviour associated with each excitation profile rather than on absolute acceleration-dependent performance. The average electrical power was calculated using (P_avg_ = V_rms_^2^/R), where (V_rms_) is the measured RMS voltage and (R) is the external load resistance. All power values reported in this work correspond to measurements obtained using a matched load resistance of 50 kΩ.

## 4. Conclusions

This work has presented a unified rotational energy harvesting architecture that enables broadband energy conversion across a wide range of angular velocities and excitation conditions by exploiting dual-mode dynamics and orientation-adaptive behaviour within a single compact structure. By integrating gravity-assisted excitation, magnetic coupling, and centripetal-force-induced nonlinear bi-stability, the proposed harvester overcomes the fundamental limitation of conventional rotational energy harvesters, which are typically restricted to narrow operating regimes. Numerical investigations demonstrated that the harvester exhibits distinct dynamic regimes as a function of angular velocity. At low rotational speeds, gravity-dominated mono-stable dynamics enable effective energy harvesting without reliance on resonance, while at elevated rotational speeds, axial loading combined with magnetic repulsion gives rise to nonlinear bi-stable behaviour characterised by large-amplitude inter-well oscillations and broadband frequency response. These numerical findings provided clear insight into the underlying nonlinear mechanisms governing the system’s behaviour. Experimental studies under controlled laboratory excitation confirmed the existence of both mono-stable and bi-stable operating regimes and showed good qualitative agreement with numerical predictions. The experiments demonstrated that gravity-driven excitation produces stable and repeatable oscillations at low excitation levels, while increased excitation activates bi-stable dynamics, leading to enhanced mechanical response and electrical output. Although practical effects such as damping, magnetic asymmetry, and alignment tolerances influenced quantitative performance, the core dual-mode operating principle was consistently observed. Further experimental investigations under realistic, non-stationary vibration environments revealed pronounced orientation-adaptive behaviour. Comparative evaluation of upward- and downward-orientated cantilever configurations showed complementary performance across diverse noise profiles, including aerospace, automotive, civil infrastructure, and industrial excitations. Time–frequency analysis confirmed that gravitational bias and inertial asymmetry play a critical role in facilitating inter-well transitions and broadband energy harvesting under high-energy, broadband excitation. Overall, the results establish gravity- and magnet-induced bi-stability combined with orientation adaptability as an effective strategy for broadband rotational energy harvesting. The proposed architecture provides a stator-independent, passive, and scalable solution for powering self-sustained sensing systems in complex rotational environments. Future work will focus on long-term durability assessment, optimisation of magnetic and structural parameters for application-specific deployment, and integration of power management circuitry to enable fully autonomous sensing platforms.

## Figures and Tables

**Table 1:** Geometric and physical parameters of the proposed rotational energy harvester.

Parameter	Value
Stainless steel cantilever dimensions	14 mm × 10 mm × 0.15 mm
PVDF dimensions	14 mm × 7 mm × 0.18 mm
Magnetic tip mass dimensions	10 mm × 10 mm × 10 mm
Free-moving magnet dimensions	10 mm × 10 mm × 10 mm
Magnet material	NdFeB (N52)
Keeper dimensions	5 mm × 12 mm × 12 mm
Guide tube dimensions	12 mm × 12 mm × 50 mm
Outer body dimensions	80 mm × 12 mm × 64 mm
Cantilever orientation	Upward and downward
Free-magnet travel direction	Vertical translation within guide tube
Initial magnetic separation distance	5 mm
External load resistance	50 kΩ
Piezoelectric material	PVDF

## Data Availability

The data presented in this study are available on request from the corresponding author. The data are not publicly available due to ongoing research work.
